# The RuvA Homologues from *Mycoplasma genitalium* and *Mycoplasma pneumoniae* Exhibit Unique Functional Characteristics

**DOI:** 10.1371/journal.pone.0038301

**Published:** 2012-05-30

**Authors:** Marcel Sluijter, Silvia Estevão, Theo Hoogenboezem, Nico G. Hartwig, Annemarie M. C. van Rossum, Cornelis Vink

**Affiliations:** Laboratory of Pediatrics, Pediatric Infectious Diseases and Immunity, Erasmus MC-Sophia Children's Hospital, Rotterdam, The Netherlands; Emory University School of Medicine, United States of America

## Abstract

The DNA recombination and repair machineries of *Mycoplasma genitalium* and *Mycoplasma pneumoniae* differ considerably from those of gram-positive and gram-negative bacteria. Most notably, *M. pneumoniae* is unable to express a functional RecU Holliday junction (HJ) resolvase. In addition, the RuvB homologues from both *M. pneumoniae* and *M. genitalium* only exhibit DNA helicase activity but not HJ branch migration activity in vitro. To identify a putative role of the RuvA homologues of these mycoplasmas in DNA recombination, both proteins (RuvA*_Mpn_* and RuvA*_Mge_*, respectively) were studied for their ability to bind DNA and to interact with RuvB and RecU. In spite of a high level of sequence conservation between RuvA*_Mpn_* and RuvA*_Mge_* (68.8% identity), substantial differences were found between these proteins in their activities. First, RuvA*_Mge_* was found to preferentially bind to HJs, whereas RuvA*_Mpn_* displayed similar affinities for both HJs and single-stranded DNA. Second, while RuvA*_Mpn_* is able to form two distinct complexes with HJs, RuvA*_Mge_* only produced a single HJ complex. Third, RuvA*_Mge_* stimulated the DNA helicase and ATPase activities of RuvB*_Mge_*, whereas RuvA*_Mpn_* did not augment RuvB activity. Finally, while both RuvA*_Mge_* and RecU*_Mge_* efficiently bind to HJs, they did not compete with each other for HJ binding, but formed stable complexes with HJs over a wide protein concentration range. This interaction, however, resulted in inhibition of the HJ resolution activity of RecU*_Mge_*.

## Introduction

A significant proportion of the genomes of *Mycoplasma pneumoniae* and *Mycoplasma genitalium* (approximately 8% and 4%, respectively) is composed of repeated DNA elements. These elements are referred to as RepMP elements in *M. pneumoniae* [1], [2], [3] and MgPa repeats (MgPars) in *M. genitalium* [4], [5], [6]. Although the different variants of these elements show a high level of sequence homology, they are not identical. Moreover, one or more of these variants are contained within open reading frames (ORFs) that encode antigenic surface proteins. Among these proteins are P1, P40 and P90 of *M. pneumoniae* and MgPa and P110 of *M. genitalium*. As these proteins can display amino acid sequence variation within the regions encoded by the RepMP and MgPar sequences, it has been proposed that this variation originates from recombination between different variants of RepMP or MgPar [7], [8],[9],[10],[11],[12],[13]. Consequently, homologous recombination between the repeated DNA elements in both *Mollicutes* species may play a crucial role in immune evasion [14].

It has previously been suggested that the mechanism of recombination between repeated DNA elements in *M. pneumoniae* and *M. genitalium* is similar to that of general homologous DNA recombination in these species [15], [16]. As a consequence, these processes may utilize the same enzymatic machinery. Recent studies that were aimed at elucidation of the mechanism of recombination between repeated DNA elements therefore focused on the characterization of *Mycoplasma* proteins predicted to be involved in homologous DNA recombination, such as RecA [15], single-stranded DNA-binding protein (SSB) [16], RuvA [17], RuvB [18] and RecU [19], [20]. The RecA proteins from *M. pneumoniae* and *M. genitalium* (RecA*_Mpn_* and RecA*_Mge_*, respectively) and the SSB protein from *M. pneumoniae* (SSB*_Mpn_*) were reported to possess similar activities as their counterparts from *Escherichia coli* [15], [16]. Both RecA*_Mpn_* and RecA*_Mge_* were found to catalyze the exchange of homologous DNA strands in an ATP- and Mg^2+^-dependent fashion [15]. This activity was stimulated strongly by SSB*_Mpn_*, which is a tetrameric protein that selectively binds to single-stranded DNA (ssDNA) [16].

In contrast to the SSB and RecA proteins, the RecU, RuvA and RuvB proteins from *M. pneumoniae* and *M. genitalium* displayed in vitro activities that differed considerably from those of their counterparts from other bacterial classes. Specifically, the RecU protein from *M. genitalium* (RecU*_Mge_*) was found to diverge from other Holliday junction (HJ) resolving enzymes in four major aspects [19]. First and foremost, RecU*_Mge_* only displayed HJ resolvase activity in the presence of Mn^2+^ and not in the presence of Mg^2+^. In contrast, the RecU homologue from *Bacillus subtilis* (RecU*_Bsu_*) and the RuvC*_Eco_* and RusA*_Eco_* resolvases from *E. coli* possess Mg^2+^-dependent resolvase activity. Second, RecU*_Mge_* has a unique target DNA sequence, cleaving HJ substrates at the sequence 5′-^G^/_T_C↓PyTPuG-3′. This cleavage site differs from the cleavage sites of RecU*_Bsu_*, RuvC*_Eco_* and RusA*_Eco_* (5′-^G^/_T_G↓C^A^/_C_-3′, 5′-^A^/_T_TT↓^G^/_C_-3′ and 5′-↓CC-3, respectively) [21], [22], [23], [24], [25]. Third, unlike the RecU*_Bsu_* protein [21], RecU*_Mge_* is unable to anneal circular ssDNA to homologous, linear double-stranded DNA (dsDNA). Fourth, RecU*_Mge_* does not stably bind to long ssDNA substrates, in contrast to the RecU*_Bsu_* protein [21].

Another crucial finding regarding the RecU orthologues from *M. pneumoniae* and *M. genitalium* was the inability of *M. pneumoniae* to produce a functional RecU protein [19], [20]. While a subset of *M. pneumoniae* strains (so-called subtype 2 strains) is able to express a RecU homologue (RecU*_Mpn_*), this protein was found to be inactive in HJ-binding and -cleavage in vitro. Moreover, the other major subset of *M. pneumoniae* strains (subtype 1 strains) was reported to be incapable of producing a full-length RecU homologue, due to the presence of a nonsense codon in the RecU gene [19]. The inability of *M. pneumoniae* to produce a functional RecU protein was suggested to be (one of) the causative factor(s) of the relatively low level of homologous DNA recombination in this bacterium [19].

Unique properties were recently also attributed to the RuvB homologues from *M. genitalium* and *M. pneumoniae* (RuvB*_Mge_* and RuvB_FH_, respectively). In contrast to the *E. coli* DNA branch migration motor protein RuvB*_Eco_*, both RuvB*_Mge_* and RuvB_FH_ were found to have RuvA-independent DNA helicase activity [18]. The activity of RuvB*_Mge_*, however, was significantly lower than that of RuvB_FH_. Interestingly, RuvB_FH_ is exclusively expressed by subtype 2 strains of *M. pneumoniae*. The RuvB protein expressed by subtype 1 strains (RuvB_M129_) displays only marginal levels of DNA helicase activity, due to a single amino acid substitution with respect to RuvB_FH_ [18]. Although RuvB_FH_ did not appear to be stimulated at all by *M. pneumoniae* RuvA (RuvA*_Mpn_*), the helicase activity of the RuvB*_Mge_* protein was found to be promoted by *M. genitalium* RuvA (RuvA*_Mge_*) under specific reaction conditions [18].

The apparent inability of RuvA*_Mpn_* to stimulate RuvB_FH_ activity can be caused by specific, aberrant features of the RuvB_FH_ protein in comparison with RuvB*_Eco_*. Alternatively, RuvA*_Mpn_* itself may be unable to interact with, and/or activate, RuvB_FH_. In this regard, it is interesting to note that RuvA*_Mpn_* did not stimulate the branch migration activity of RuvB*_Eco_ i*n vitro, and could not functionally substitute for RuvA*_Eco_* in vivo (in *E. coli*) [17]. Thus, while the *E. coli* RuvA protein has a vital role in the interaction with both RuvB and the HJ resolving enzyme RuvC (within the RuvABC resolvasome), the function of RuvA*_Mpn_* within a putative branch migration and resolution complex remains enigmatic.

In this study, the activities of RuvA*_Mpn_* and RuvA*_Mge_* are characterized and compared. We show that these proteins differ considerably in (i) their affinities for branched and non-branched DNA substrates, (ii) complex formation with HJs, and (ii) their interaction with other proteins from the DNA recombination machinery.

## Results

### *M. pneumoniae* ORF MPN535 and *M. genitalium* ORF MG358 encode RuvA homologues

**Figure 1:**
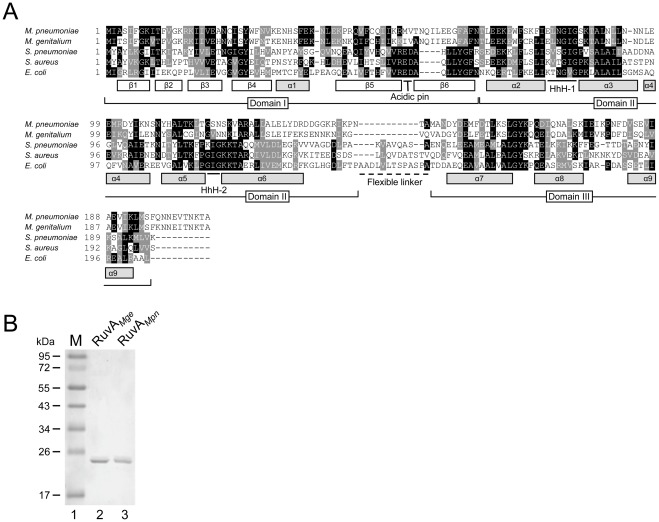
Multiple alignment and purification of RuvA*_Mpn_* and RuvA*_Mge_*. (A) An alignment was generated with amino acid sequences predicted to be encoded by the following ORFs (with GenBank accession numbers in parentheses), *M. pneumoniae* MPN535 (P75243), *M. genitalium* G37 MG358 (Q49424), *Streptococcus pneumoniae ruvA* (Q97SY4), *Staphylococcus aureus ruvA* (Q5HFC1) and *E. coli ruvA* (P0A809). Predicted secondary structural features and domains of the RuvA proteins are shown below the alignment and are based on the crystal structure of the RuvA protein from *E. coli* [27], [28], [30], [33], [54]. The position of the ‘acidic pin’, between β sheets 6 and 7 of RuvA*_Eco_*, two helix-hairpin-helix (HhH) motifs, and the flexible linker (between domain II and III), are also indicated. The multiple alignment was performed using Clustal W (http://www.ebi.ac.uk/Tools/msa/clustalw2/). The program BOXSHADE 3.21 (http://www.ch.embnet.org/software/BOX_form.html) was used to generate white letters on black boxes (for residues that are identical in at least three out of five sequences) and white letters on grey boxes (for similar residues). (B) Purification of RuvA*_Mge_* and RuvA*_Mpn_*. Samples of purified H_10_-tagged RuvA*_Mge_* (lane 2) and H_10_-tagged RuvA*_Mpn_* (lane 3) were analyzed by SDS-PAGE (12%) and Coomassie brilliant blue (CBB)-staining. The sizes of protein markers (lane 1; PageRuler^TM^ Prestained Protein Ladder [Fermentas]) are shown on the left-hand side of the figure in kDa.

The MPN535 ORF of *M. pneumoniae* was previously shown to encode a RuvA homologue (RuvA*_Mpn_*) [17]. A multiple amino acid sequence alignment indeed shows significant similarities between RuvA*_Mpn_* and other (putative) RuvA proteins from gram-negative and gram-positive bacteria (Fig. 1A). While the similarity between the sequences of RuvA*_Mpn_* and RuvA*_Eco_* is relatively low (23.6% identity), a high similarity is observed between the sequences of RuvA*_Mpn_* and RuvA*_Mge_* (68.8% identity). In contrast to other members of the putative DNA recombination apparatus of *M. pneumoniae*, i.e. RecU and RuvB [18], [19], RuvA*_Mpn_* does not differ in sequence among subtype 1 and subtype 2 strains.

Within the RuvA sequences, a relatively high level of amino acid sequence conservation is found in two so-called helix-hairpin-helix (HhH) motifs (Fig. 1A) [26]. These motifs were previously identified within domain II of RuvA*_Eco_* and were shown to be crucial for sequence-independent DNA binding by interacting with the DNA phosphate backbone of Holliday junctions (HJs) [27], [28], [29]. The lowest level of sequence conservation was seen in the region defined as the ‘flexible linker’, which separates domain II from domain III in RuvA*_Eco_* [30].

### RuvA*_Mge_* and RuvA*_Mpn_* can bind to synthetic oligonucleotide substrates

Both RuvA*_Mge_* and RuvA*_Mpn_* were expressed in *E. coli* as poly histidine (H_10_)-tagged proteins and were purified to near homogeneity using similar protocols (as described in Materials and Methods). The H_10_-tagged proteins were found to have activities that were indistinguishable from that of their non-tagged counterparts (data not shown). Because the H_10_-tagged proteins were obtained at higher concentrations and at a higher purity than their ‘native’ versions (>95% versus ∼90% homogeneity), they were used throughout this study. The estimated molecular masses of the purified proteins matched the theoretical molecular masses of 23.7 kDa for both RuvA*_Mge_* (Fig. 1B, lane 2) and RuvA*_Mpn_* (lane 3).

**Figure 2:**
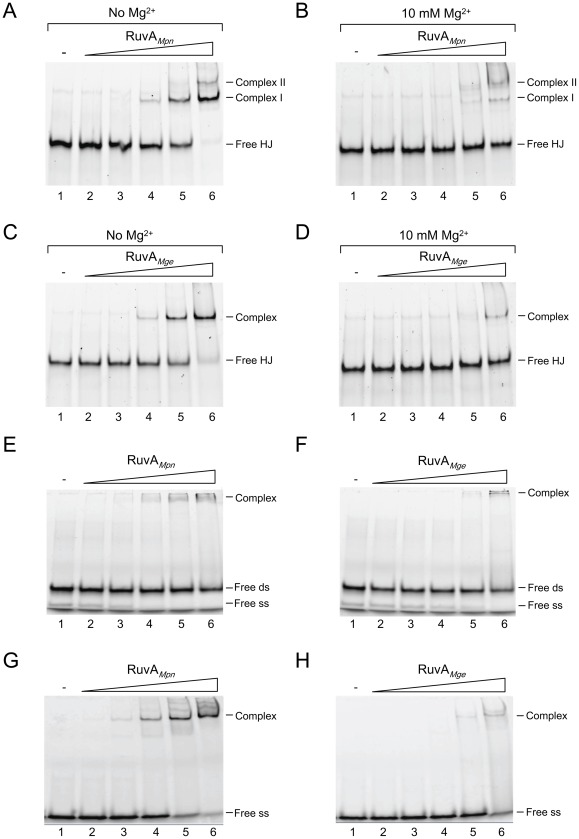
Binding of RuvA*_Mpn_* and RuvA*_Mge_* to HJs and other oligonucleotide substrates. (A) Binding of RuvA*_Mpn_* to HJ substrate HJ 1.1 in the absence of Mg^2+^. The DNA-binding reactions were performed as indicated in Materials and Methods. Reactions were performed in volumes of 10 μl and contained 12.3 nM DNA substrate and either 0 nM (marked ‘-’, lane 1), 27 nM (lane 2), 81 nM (lane 3), 243 nM (lane 4), 729 nM (lane 5) or 2.2 μM (lane 6) of RuvA*_Mpn_*. Reaction products were electrophoresed through 8% polyacrylamide gels and analyzed by fluorometry. The positions of unbound HJ 1.1 (Free HJ) and RuvA*_Mpn_*/HJ complexes (Complex I and II) are indicated at the right-hand side of the gel. (B) Binding of RuvA*_Mpn_* to HJ substrate HJ 1.1 in the presence of 10 mM Mg^2+^. Reactions were carried out in a similar fashion as in (A). (C, D). Binding of RuvA*_Mge_* to HJ substrate HJ 1.1 in the absence (C) or presence of 10 mM Mg^2+^ (D). (E, F) Binding of RuvA*_Mpn_* (E) and RuvA*_Mge_* (F) to double-stranded (ds) oligonucleotide HJ11/HJ11rv. The positions of the ds substrate (Free ds) and residual non-annealed oligonucleotide HJ11 (Free ss) is indicated at the right-hand side of the gels. (G, H) Binding of RuvA*_Mpn_* (G) and RuvA*_Mge_* (H) to single-stranded (ss) oligonucleotide HJ11. The reactions shown in panels (C) to (H) were carried out similarly as in (A).

To test and compare the DNA-binding characteristics of RuvA*_Mge_* and RuvA*_Mpn_*, both proteins were incubated with HJs, double-stranded (ds) and single-stranded (ss) oligonucleotide substrates, and analyzed by electrophoretic mobility shift assay (EMSA). As described before [17], two distinct complexes (complex I and complex II) were formed between RuvA*_Mpn_* and HJs in a protein-concentration dependent fashion (Fig. 2A). Similar complexes were reported to be generated between RuvA*_Eco_* and HJs, and were found to consist of a single protein tetramer (complex I) or a double tetramer (complex II) bound to a HJ [30], [31], [32], [33], [34], [35]. The HJ binding activity of both RuvA*_Mpn_* and RuvA*_Mge_* was strongly reduced in the presence of Mg^2+^ (compare Fig. 2A to Fig. 2B, and Fig. 2C to Fig. 2D). A similar inhibitory effect of Mg^2+^ on DNA-binding activity has previously also been observed for RuvA*_Eco_* [31], [36]. In contrast to RuvA*_Eco_* and RuvA*_Mpn_*, RuvA*_Mge_* produced only a single complex with HJs (Fig. 2C, lane 6), even at protein concentrations up to 4 μM (see below). This complex migrated through the gels with a mobility similar to that of RuvA*_Mpn_*-HJ complex I. These data indicated that: (i) the RuvA*_Mge_*-HJ complex is composed of a tetramer of RuvA*_Mge_* bound to a HJ, and (ii) RuvA*_Mge_* may not stably bind to HJs as an octamer. These notions were supported by gel filtration chromatography data, which indicated that RuvA*_Mge_* exists as a single, major protein species with a molecular mass of ∼108 kDa (Fig. S1). This molecular mass corresponds to the theoretical molecular mass of a tetramer of RuvA*_Mge_* (95 kDa). Thus, RuvA*_Mge_* primarily exists as a homo-tetramer in solution.

In contrast to RuvA*_Eco_*, RuvA*_Mpn_* was previously reported to form stable complexes with linear duplex oligonucleotides [17]. As shown in Fig. 2E and 2F, both RuvA*_Mpn_* and RuvA*_Mge_* are able to form DNA-protein complexes in the presence of ds oligonucleotides (substrate HJ11/HJ11rv). Interestingly, at least part of these complexes consisted of RuvA molecules bound to non-annealed, ss oligonucleotide HJ11, which was present as a minor ‘contaminant’ of the ds substrate; this oligonucleotide (designated ‘Free ss’ in Fig. 2E and 2F) was completely complexed by the RuvA proteins at the highest protein concentrations tested (Fig. 2E, lane 4–6 and Fig. 2F, lane 6). In a separate EMSA, we could confirm the binding of RuvA*_Mpn_* to oligonucleotide HJ11; this binding appeared to occur with an efficiency similar to that observed with the four-stranded HJ substrate (compare Fig. 2G to Fig. 2A). Conversely, while the RuvA*_Mge_* protein also displayed binding to the ssDNA (Fig. 2H), this binding was considerably less efficient than that observed with the HJ substrate (Fig. 2C).

**Figure 3:**
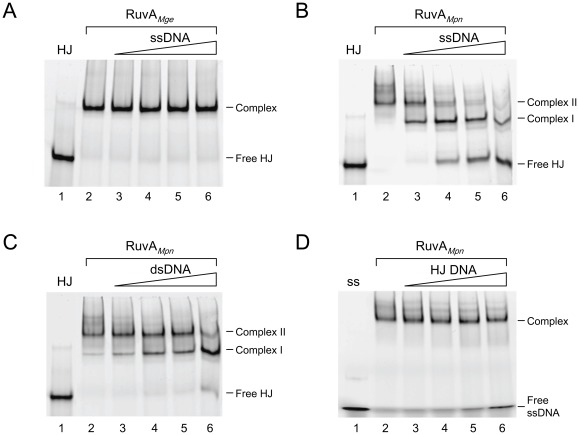
DNA binding preferences of RuvA*_Mge_* and RuvA*_Mpn_*. (A) Binding of RuvA*_Mge_* (3 µM) to HJ substrate HJ 1.1 (6-FAM-labeled on strand HJ11) in the presence of various concentrations of unlabeled ssDNA (oligonucleotide HJ11). The molar excess of unlabeled DNA over labeled DNA in the reactions was 0× (lane 2), 2.5× (lane 3), 5× (lane 4), 10× (lane 5) and 20× (lane 6). The protein was added as final component in the reactions. Protein was omitted from the reaction shown in lane 1. The positions of the free HJ substrate (Free HJ) and RuvA*_Mge_*-HJ complexes (Complex) are indicated at the right-hand side of the gel. (B) Binding of RuvA*_Mpn_* (3 µM) to HJ substrate HJ 1.1 (6-FAM-labeled on strand HJ11) in the presence of various concentrations of unlabeled ssDNA (oligonucleotide HJ11). The experiment was performed similarly as in (A). The two major RuvA*_Mpn_*-HJ complexes (Complex I and II) are indicated at the right-hand side of the gel. (C) Binding of RuvA*_Mpn_* (3 µM) to HJ substrate HJ 1.1 (6-FAM-labeled on strand HJ11) in the presence of various concentrations of unlabeled dsDNA (oligonucleotide HJ11/HJ11rv). The experiment was performed similarly as in (A). The two major RuvA*_Mpn_*-HJ complexes (Complex I and II) are indicated at the right-hand side of the gel. (D) Binding of RuvA*_Mpn_* (3 µM) to ssDNA (6-FAM-labeled oligonucleotide HJ11) in the presence of various concentrations of HJ DNA (HJ 1.1). The experiment was performed similarly as in (A). Protein was omitted from the reaction shown in lane 1. The positions of the unbound ssDNA (Free ssDNA) and RuvA*_Mpn_*-ssDNA complex (Complex) are indicated at the right-hand side of the gel.

The preferences of RuvA*_Mpn_* and RuvA*_Mge_* for binding to either ssDNA or HJ DNA were further investigated in DNA-binding competition experiments, in which a labeled DNA substrate was kept at a constant concentration and another, unlabeled substrate was included at different concentrations. As shown in Fig. 3A, the binding of RuvA*_Mge_* to the labeled HJ substrate was not significantly influenced by inclusion of up to a 20-fold excess of unlabeled ssDNA in the reaction (lanes 3–6). In contrast, the binding of RuvA*_Mpn_* to the HJ substrate was already clearly reduced in the presence of a 2.5-fold excess of unlabeled ssDNA in the binding reactions (Fig. 3B, lane 3). Although the dsDNA substrate also competed with the HJ substrate for binding by RuvA*_Mpn_*, this competition was less efficient than that observed with ssDNA (Fig. 3C). The high affinity of RuvA*_Mpn_* for ssDNA was further demonstrated in an experiment in which the binding of RuvA*_Mpn_* to labeled ssDNA was assayed in the presence of different concentrations of unlabeled HJ substrate. As shown in Fig. 3D, the ssDNA-binding of RuvA*_Mpn_* was only marginally reduced in the presence of a 10-fold (lane 5) or 20-fold (lane 6) molar excess of unlabeled HJ DNA in the reactions. Thus, in contrast to RuvA*_Mge_* (Fig. 2H and 3A), RuvA*_Mpn_* is able to bind with a relatively high affinity to ssDNA.

### The interaction between RuvA*_Mge_* and RecU*_Mge_* on HJs

**Figure 4:**
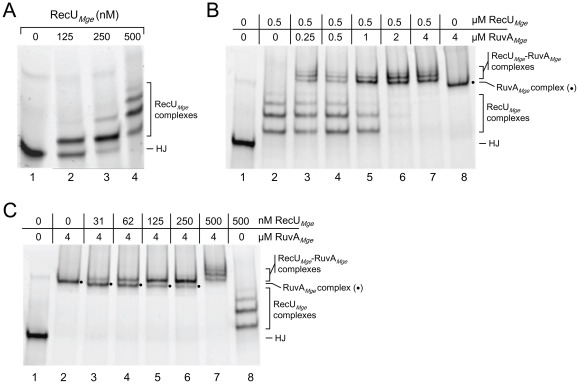
The interaction between RuvA*_Mge_* and RecU*_Mge_* on HJs. (A) HJ-binding by RecU*_Mge_*. The DNA-binding reactions were performed in a similar fashion as described in Fig. 3. Reactions were performed in volumes of 10 μl and contained 12.3 nM HJ 1.1 and the indicated concentrations of RecU*_Mge_*. The positions of unbound HJs (HJ) and RecU*_Mge_*-HJ complexes are depicted at the right-hand side of the gel. (B) The binding of RuvA*_Mge_* to RecU*_Mge_*-HJ complexes. RecU*_Mge_* (0.5 μM) was incubated with HJ 1.1, followed by the addition of RuvA*_Mge_* (at different concentrations, as indicated above the lanes). The nature of the various protein-DNA complexes is indicated at the right-hand side of the gel; RuvA*_Mge_*-HJ complexes are indicated with a dot (•). (C) The binding of RecU*_Mge_* to RuvA*_Mge_*-HJ complexes. RuvA*_Mge_* (4 μM) was incubated with HJ 1.1, followed by the addition of RecU*_Mge_* (at various concentrations, as indicated above the lanes). The labeling of the figure is similar to that shown in (B).

The RecU protein from *M. pneumoniae* (RecU*_Mpn_*) was previously found to be inactive in HJ-binding and -cleavage [19]. In contrast, the *M. genitalium* RecU protein (RecU*_Mge_*) was reported to be a potent HJ-resolving enzyme [19], [20]. Because it is possible that RecU*_Mge_* functionally interacts with RuvA*_Mge_* in the processing of HJs, both proteins were included in HJ binding and resolution assays. The binding of RecU*_Mge_* to HJ substrate HJ 1.1 was previously demonstrated to result in a single DNA-protein complex [19], [20]. Interestingly, at relatively high RecU*_Mge_* concentrations and at different binding conditions than those used previously (i.e., binding on ice instead of at room temperature and in the absence of BSA), a range of discrete RecU*_Mge_*-HJ DNA-protein complexes were generated, with an inverse correlation between protein concentration and mobility of the complexes through EMSA gels (Fig. 4A, lanes 2–4). At 500 nM of RecU*_Mge_*, three major DNA-protein complexes and one minor complex can be discerned (lane 4). A similar range of complexes was previously also observed after binding of *E. coli* resolvase RusA to HJ substrates [25]. Due to the distinct nature of the RecU*_Mge_*-HJ complexes and their relative migration in the gel, we hypothesize that they represent different multimeric forms of RecU*_Mge_*, bound to a single HJ substrate. Upon addition of RuvA*_Mge_* to these complexes (after preincubation of RecU*_Mge_* with the HJ substrate), novel complexes were formed with a considerably slower mobility than the RecU*_Mge_*-HJ complexes (Fig. 4B, lanes 3–7). At the highest concentration of RuvA*_Mge_* used (4 μM), all RecU*_Mge_*-HJ complexes appeared to have shifted to a higher position in the gel (lane 7). Because the novel complexes had a slower mobility than the RuvA*_Mge_*-HJ complex (Fig. 4B, lane 8), it is likely that they represent HJs bound by both RecU*_Mge_* and RuvA*_Mge_*. This notion was corroborated by a reciprocal experiment in which the HJ substrate was preincubated with RuvA*_Mge_* (at 4 μM), followed by the addition of RecU*_Mge_* at concentrations ranging from 0 nM to 500 nM (Fig. 4C, lanes 2–7). Already at a RecU*_Mge_* concentration of 31 nM (lane 3), a ‘supershift’ of the RuvA*_Mge_*-HJ complex was observed; this supershift was virtually complete at a RecU*_Mge_* concentration of 250 nM (lane 6). At the latter concentration, a single major supershifted complex was observed. At 500 nM of RecU*_Mge_*, however, four discrete supershifted complexes were formed, which corresponded in mobility with the complexes generated in the previous experiment (Fig. 4B, lane 7). Again, the supershifted complexes displayed a slower mobility than did the RecU*_Mge_*-HJ and RuvA*_Mge_*-HJ complexes (Fig. 4C, lanes 2 and 8), indicating that they indeed represent RecU*_Mge_*-RuvA*_Mge_*-HJ complexes. The interactions between RecU*_Mge_* and RuvA*_Mge_* on HJ substrates differ significantly from those reported between RuvA*_Eco_* and the RuvC resolvase from *E. coli* (RuvC*_Eco_*). Specifically, RuvA*_Eco_* appears to have a significantly higher affinity than RuvC*_Eco_* for HJ substrates, and a fully saturated RuvA*_Eco_*-HJ complex (complex II) cannot be bound detectably by RuvC*_Eco_* [32]. As a consequence, RuvAC*_Eco_*-HJ complexes are only observed at relatively low RuvA*_Eco_* concentrations (1–20 nM); at higher RuvA*_Eco_* concentrations, RuvAC*_Eco_*-HJ and RuvC*_Eco_*-HJ complexes are either not formed or rapidly dissociated [32]. In contrast, RecU*_Mge_* and RuvA*_Mge_* do not appear to compete with each other in HJ binding, but rather associate readily and stably on a HJ substrate at a wide range of concentrations of both RuvA*_Mge_* (Fig. 4B) and RecU*_Mge_* (Fig. 4C). As yet, the multimeric protein composition of the different RecU*_Mge_*-RuvA*_Mge_*-HJ complexes is unknown. Nevertheless, while a single stable complex is generated between RuvA*_Mge_* and HJs, it is likely that each of the RecU*_Mge_*-RuvA*_Mge_*-HJ complexes only contains a single tetramer of RuvA*_Mge_*.

### RuvA*_Mge_* inhibits HJ resolution by RecU*_Mge_*

**Figure 5:**
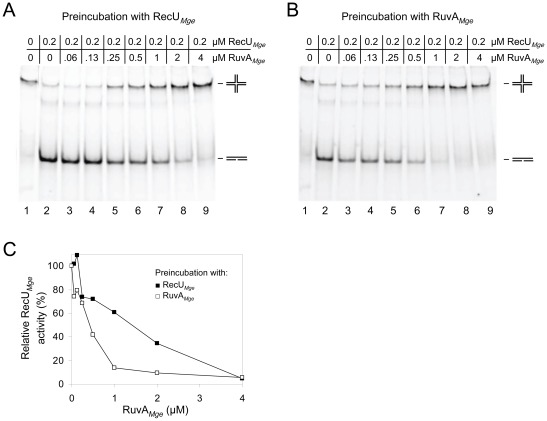
RuvA*_Mge_* inhibits HJ resolution by RecU*_Mge_*. (A, B) HJ resolution assays [19] were performed in volumes of 10 µl and contained 12.3 nM HJ substrate HJ 1.1 (6-FAM-labeled), RecU*_Mge_* (0.2 µM) and various concentrations of RuvA*_Mge_*, as indicated above the lanes. Reactions were preincubated for 2 min with either RecU*_Mge_* (A) or RuvA*_Mge_* (B), followed by addition of the other protein. After incubation for 30 min at 37°C, the reaction products were separated on 12% polyacrylamide gels, and analyzed by fluorometry. The locations of the HJ substrate and resolution products are indicated schematically at the right-hand side of the gels. (C) Quantification of the influence of RuvA*_Mge_* on RecU*_Mge_* activity. The relative RecU*_Mge_* (resolution) activity was measured from the gels shown in (A) and (B) and expressed as percentage of the protein's activity in the absence of RuvA*_Mge_*. The data from (A) and (B) are represented by the closed squares (▪) and the open squares (□), respectively.

Because RuvA*_Mge_* readily binds to RecU*_Mge_*-HJ complexes, we investigated the influence of RuvA*_Mge_* on the activity of RecU*_Mge_* in HJ resolution assays. In these assays, substrate HJ 1.1 was preincubated on ice with either RecU*_Mge_* (at 0.2 μM; Fig. 5A) or RuvA_Mge_ (at 0 to 4 μM; Fig. 5B), followed by the addition of the other protein. After incubation for 30 min at 37°C, the resolution products were analyzed by polyacrylamide gel electrophoresis. As shown in Fig. 5A and 5B, RuvA*_Mge_* inhibited the resolution activity of RecU*_Mge_* in a RuvA*_Mge_* concentration-dependent fashion. The inhibition of HJ resolution was most effective when RuvA*_Mge_* was added to the HJ substrate before RecU*_Mge_* (Fig. 5B and 5C). In that case, HJ resolution by RecU*_Mge_* was already inhibited by ∼20% at a RuvA*_Mge_* concentration of 60 nM (Fig. 5B, lane 3 and Fig. 5C). At RuvA*_Mge_* concentrations of 1 μM or higher, RecU*_Mge_* activity was reduced by ≥80% (Fig. 5B, lanes 7–9). When the HJ substrate was incubated with RecU*_Mge_* before the addition of RuvA*_Mge_*, a significant inhibition of HJ resolution activity (≥20%) was only observed at RuvA*_Mge_* concentrations of ≥250 nM (Fig. 5A and 5C). Moreover, inhibition levels of >80% were not observed at RuvA*_Mge_* concentrations lower than 4 μM. When RecU*_Mge_* and RuvA*_Mge_* were added simultaneously to the HJ substrates, a similar pattern of HJ resolution was observed as that shown in Fig. 5A (in which RecU*_Mge_* was added to the reactions before RuvA*_Mge_*). This finding corroborates the notion that RecU*_Mge_*-HJ complexes cannot be dissociated by RuvA*_Mge_*. Despite the significantly different dynamics in the formation of RecU*_Mge_*-RuvA*_Mge_*-HJ complexes and RuvAC*_Eco_*-HJ complexes, RuvA*_Mge_* inhibits the resolution activity of RecU*_Mge_* in a similar fashion as RuvA*_Eco_* inhibits RuvC*_Eco_* activity.

### The influence of the RuvA proteins on the activities of RuvB_FH_ and RuvB*_Mge_*

**Figure 6:**
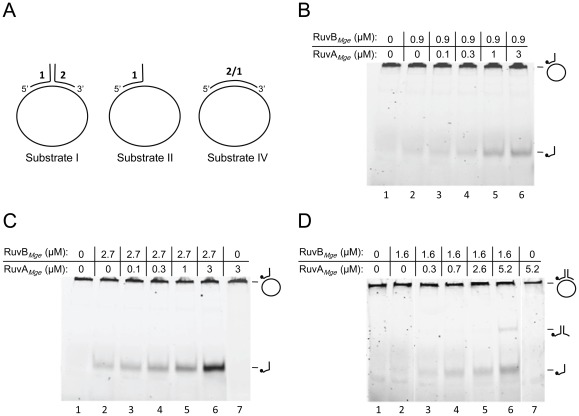
The influence of RuvA*_Mge_* on the DNA helicase activity of RuvB*_Mge_*. (A) Schematic illustrations of the DNA substrates used in the DNA helicase assays. The substrates are composed of a combination of oligonucleotides (oligonucleotide 1, oligonucleotide 2 or oligonucleotide 2/1) and single-stranded, circular 5,386-bp φX174 DNA, as described previously [18]. (B, C) RuvA*_Mge_* stimulates the DNA helicase activity of RuvB*_Mge_* on Substrate II. Substrate II, 6-FAM-labeled at the 5′ end of oligonucleotide 1, was incubated with either 0 µM, 0.9 µM (B) or 2.7 µM (C) of RuvB*_Mge_* in the presence of various concentrations of RuvA*_Mge_*, as indicated above the lanes. (D) RuvA*_Mge_* stimulates the DNA helicase activity of RuvB*_Mge_* on Substrate I. Substrate I, 6-FAM-labeled at the 5′ end of oligonucleotide 1, was incubated with either 0 µM (lanes 1 and 7) or 1.6 µM of RuvB*_Mge_* in the presence of various concentrations of RuvA*_Mge_*, as indicated above the lanes. After the reaction (5 min at 37°C), the samples were deproteinized, electrophoresed through native 12% polyacrylamide gels, and analyzed by fluorometry. The positions of the substrates, which are too large to enter the gels, as well as the positions of the oligonucleotide reaction products, are indicated at the right-hand side of the gels by schematic illustrations. In these illustrations, the position of the 6-FAM label is indicated by a black dot.

The RuvB protein that is expressed by *M. pneumoniae* subtype 2 strains, RuvB_FH_, was recently reported to act as a DNA helicase on specific, partially double-stranded DNA substrates [18]. Interestingly, while this activity of RuvB_FH_ was not influenced by RuvA*_Mpn_*, the RuvB protein from *M. genitalium*, RuvB*_Mge_*, did show RuvA*_Mge_*-dependent helicase activity. The latter activity, however, was only detected on a single helicase substrate, i.e. Substrate IV from Fig. 6A [18]. To further delineate the functional interactions between the RuvA and RuvB proteins from *M. pneumoniae* and *M. genitalium*, the proteins were combined at various concentrations (including considerably higher RuvA concentrations than used previously) in DNA helicase or branch migration assays, using the DNA helicase substrates shown in Fig. 6A. While the helicase activity of RuvB_FH_ was not influenced by RuvA*_Mpn_* (data not shown), the helicase activity of RuvB*_Mge_* on Substrate II (Fig. 6B and 6C) and Substrate I (Fig. 6D) was stimulated in the presence of high concentrations of RuvA*_Mge_*. As expected, RuvA*_Mge_* alone did not display any DNA helicase activity (lane 7 in Fig. 6C and 6D). This stimulatory effect of RuvA*_Mge_* was observed at various concentrations of RuvB*_Mge_*, from 0.9 μM (Fig. 6B) to 2.7 μM (Fig. 6C). These results indicated that the activation of RuvB*_Mge_* by RuvA*_Mge_* is a general phenomenon that is not restricted to a specific DNA substrate. Nevertheless, irrespective of the presence of high concentrations of the RuvA proteins, both RuvB*_Mge_* and RuvB*_Mpn_* were unable to unwind small, double-stranded oligonucleotide substrates (data not shown).

### The ATPase activity of RuvB*_Mge_* is stimulated by RuvA*_Mge_*

**Figure 7:**
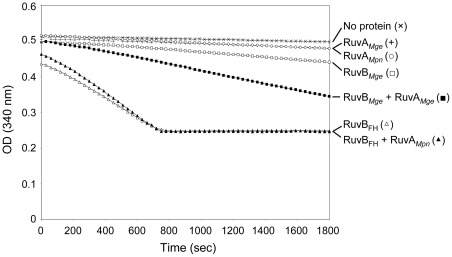
The influence of RuvA*_Mpn_* and RuvA*_Mge_* on the ATPase activities of RuvB_FH_ and RuvB*_Mge_*, respectively. ATP hydrolysis by RuvB_FH_ and RuvB*_Mge_* was measured at a protein concentration of 0.5 µM, either in the absence of presence of the corresponding RuvA protein (at 1 µM). The ATPase activity was determined using an NADH-coupled assay. In this assay, the activity is calculated from the stationary velocities of ATP hydrolysis, as determined by monitoring the absorption of NADH at 340 nm [15], [46]. The ‘no protein’ reaction (×) indicates a control reaction performed in the absence of any protein. (+), RuvA*_Mge_* alone; (○), RuvA*_Mpn_* alone; (□), RuvB*_Mge_* alone; (▪), RuvB*_Mge_* plus RuvA*_Mge_*; (▵), RuvB_FH_ alone; (▴), RuvB_FH_ plus RuvA*_Mpn_*. The graph shows a representative experiment.

While RuvB_FH_ and RuvB*_Mge_* were previously found to possess intrinsic ATPase activity, this activity was significantly higher for RuvB_FH_ than for RuvB*_Mge_* [18]. To investigate whether the ATPase activities of the RuvB proteins can be modulated by their corresponding RuvA proteins, ATPase assays were carried out in which the RuvA and RuvB proteins were tested together. In accordance with previous findings [18], RuvB_FH_ was found to possess a significantly higher ATPase activity than RuvB*_Mge_* (Fig. 7). However, while the activity of RuvB_FH_ was not significantly influenced by RuvA*_Mpn_*, the activity of RuvB*_Mge_* was strongly stimulated by RuvA*_Mge_*. Thus, the ATPase activities of RuvB_FH_ and RuvB*_Mge_* directly reflect the DNA helicase activities of these proteins in two important aspects. First, the intrinsic enzymatic activity of RuvB_FH_ is higher than that of RuvB*_Mge_*. Second, RuvB*_Mge_* activity can be stimulated by RuvA*_Mge_*, whereas RuvB_FH_ activity is not influenced by RuvA*_Mpn_*. As expected, both RuvA*_Mpn_* and RuvA*_Mge_* did not show any ATPase activity on their own (Fig. 7).

## Discussion

The DNA recombination and repair machineries of mycoplasmas differ considerably from those of gram-positive and gram-negative bacteria. Most importantly, in contrast to the latter micro-organisms, mycoplasmas do not possess homologues of LexA, RecBCD, AddAB, RecQ, RecJ and RecF [37], [38]. In addition, some components of the putative DNA recombination machineries of *M. pneumoniae* and *M. genitalium* were found to have characteristics that diverge from those of their homologues from other bacterial classes. These components include the RecU and RuvB proteins [18], [19], [20]. In Table 1, the characteristics of these as well as the other (putative) components of the DNA recombination machineries of *M. pneumoniae* and *M. genitalium* are listed and compared.

**Table 1:** Compilation of the activities of the RecA, SSB, RuvA, RuvB and RecU proteins from *M. pneumoniae*, *M. genitalium* and reference bacteria.

Protein		Species	ORF	Activities (in vitro)	Divalent cations, nucleotide and protein cofactors	Interacting proteins (physical and/or functional)	Reference
**RecA**							
	RecA*_Eco_*	*E. coli*	*recA*	Exchange of homologous DNA strands	Mg^2+^, ATP, SSB*_Eco_*	SSB*_Eco_*	[47]
	RecA*_Mpn_*	*M. pneumoniae*	MPN490	Exchange of homologous DNA strands	Mg^2+^, ATP, SSB*_Mpn_*	SSB*_Mpn_*	[15]
	RecA*_Mge_*	*M. genitalium*	MG339	Exchange of homologous DNA strands	Mg^2+^, ATP, SSB^3^	SSB^3^	[15]
**SSB**							
	SSB*_Eco_*	*E. coli*	*ssb*	ssDNA-binding, stimulation of RecA*_Eco_*, various other roles in DNA replication, repair, and recombination	None	RecA*_Eco_*, other proteins	[48]
	SSB*_Mpn_*	*M. pneumoniae*	MPN229	ssDNA-binding, stimulation of RecA*_Mpn_*	None	RecA*_Mpn_*	[16]
	SSB*_Mge_*	*M. genitalium*	MG091	Unknown	Unknown	Unknown	n.a.
**RuvA**							
	RuvA*_Eco_*	*E. coli*	*ruvA*	HJ-binding, stimulation of RuvB*_Eco_,* inhibition of RuvC*_Eco_*	None	RuvB*_Eco_*, RuvC*_Eco_*	[31], [32]
	RuvA*_Mpn_*	*M. pneumoniae*	MPN535	HJ- and ssDNA-binding	None	Unknown	This study; [17]
	RuvA*_Mge_*	*M. genitalium*	MG358	HJ-binding, stimulation of RuvB*_Mge_*, inhibition of RecU*_Mge_*	None	RuvB*_Mge_*, RecU*_Mge_*	This study
**RuvB**							
	RuvB*_Eco_*	*E. coli*	*ruvB*	HJ branch migration, DNA unwinding	Mg^2+^, ATP, RuvA*_Eco_*	RuvA*_Eco_*, RuvC*_Eco_*	[49], [50], [51]
	RuvB_FH_ 1	*M. pneumoniae*	MPN536	DNA unwinding	Mg^2+^, ATP	Unknown	This study; [18]
	RuvB*_Mge_*	*M. genitalium*	MG359	DNA unwinding	Mg^2+^, ATP, RuvA*_Mge_*	RuvA*_Mge_*	This study; [18]
**RecU**							
	RecU*_Bsu_*	*Bacillus subtilis*	*recU*	HJ resolution, annealing of homologous DNA substrates, modulation of RecA function	Mg^2+^	RuvB*_Bsu_*, RecA*_Bsu_*	[21], [52], [53]
	RecU*_Mpn_* 2	*M. pneumoniae*	MPN528a	None	n.a.	Unknown	[19]
	RecU*_Mge_*	*M. genitalium*	MG352	HJ resolution	Mn^2+^	RuvA*_Mge_*	This study; [19], [20]

1 The RuvB_FH_ protein is exclusively expressed by subtype 2 strains of *M. pneumoniae*. Subtype 1 strains express a RuvB protein (RuvB_M129_) that differs in a single amino acid residue from RuvB_FH_. RuvB_M129_ has significantly lower ATPase and DNA helicase activities than RuvB_FH_ [18].

2 RecU*_Mpn_* is only expressed by subtype 2 strains of *M. pneumoniae*. Subtype 1 strains are unable to express a full-length RecU protein due to the presence of a nonsense mutation in the RecU gene (MPN528a) [19].

3 The SSB-dependence of the RecA*_Mge_* protein was determined using SSB*_Mpn_* and SSB*_Eco_* [15]. The SSB*_Mge_* protein has not yet been characterized.

n.a., not applicable.

We here report that the RuvA proteins from both *Mycoplasma* spp., RuvA*_Mge_* and RuvA*_Mpn_*, also possess exceptional properties as opposed to their well-characterized counterpart from *E. coli*, RuvA*_Eco_*. While both RuvA*_Mge_* and RuvA*_Eco_* [31], [39] preferentially bind to HJs, RuvA*_Mpn_* displayed a high affinity for both HJ and ssDNA. In addition, while RuvA*_Mpn_* and RuvA*_Eco_* are both able to form two distinct complexes with HJ substrates, RuvA*_Mge_* only formed a single complex with HJs. As this RuvA*_Mge_*-HJ complex had a similar mobility through polyacrylamide gels as RuvA*_Mpn_*-HJ complex I and RuvA*_Eco_*-HJ complex I [17], [30], [31], [32], [33], [34], [35], and because RuvA_Mge_ is a tetramer in solution, it is highly likely that this complex is composed of a tetramer of RuvA*_Mge_* bound to a single HJ. This implies that RuvA*_Mge_* may only stably bind to HJs as a tetramer. This notion can have important consequences for the interaction of the RuvA*_Mge_*-HJ complex with other proteins that are potentially targeted to HJs, such as RuvB*_Mge_* and RecU*_Mge_*. It was previously reported that the ability of RuvA*_Eco_* to form stable octamers on HJs was vital for full activity of the protein. This notion was inferred from the activities of four different octamerization-deficient RuvA*_Eco_* mutants [34], [35], [40]. Three of these mutants carried amino acid substitutions in a protein region known to be involved in tetramer-tetramer interactions [34], [35], [40]. This region was identified within the crystal structure of HJ-bound octamers of the *Mycobacterium leprae* RuvA protein (RuvA*_Mle_*) [33]. Within this structure, the two RuvA tetramers make direct protein-protein contacts through specific amino acid side chain interactions at four equivalent points, which are localized to the α6 helix of domain II (Fig. 1A). The interacting α6 helices from two RuvA monomers are in an antiparallel configuration, such that ion pair interactions are formed between three pairs of amino acid residues. On the basis of sequence alignments, we predict that only two of such pairs may be formed between two antiparallel α6 helices of both RuvA*_Mge_* and RuvA*_Mpn_*. In RuvA*_Mpn_*, these pairs would consist of Lys121-Asp133 and Arg124-Glu130, whereas in RuvA*_Mge_*, they would consist of Lys121-Glu133 and Arg124-Glu130. While this prediction emphasizes the sequence similarity between RuvA*_Mpn_* and RuvA*_Mge_*, it does not provide an explanation why RuvA*_Mpn_* is able to form stable octameric complexes with HJs, and RuvA*_Mge_* is not. It should be considered, however, that the octamerization signals of RuvA*_Mpn_* (which are absent from RuvA*_Mge_*) may differ considerably from those of RuvA*_Mle_*, and are not (solely) determined by contacts between amino acid residues located in the α6 helix. In this regard, it is relevant to note that one of the reported RuvA*_Eco_* mutants that is unable to form stable octamers on HJs, RuvAz87, does not carry mutations in helix α6, but in two other regions of the protein, *i.e.* in the region between helices α2 and α3 and in helix α4 [40].

Despite its inability to octamerize on HJs in a stable fashion, RuvA*_Mge_* was found to stimulate the DNA helicase and ATPase activities of RuvB*_Mge_*. The octamerization-competent RuvA*_Mpn_* protein, however, did not augment RuvB_FH_ activity. It is possible that the relatively high intrinsic DNA helicase activity of RuvB_FH_ obscured the observation of any additional stimulatory effect on this protein by RuvA*_Mpn_*. An alternative explanation for the inability of RuvA*_Mpn_* to boost RuvB_FH_ activity is that these proteins are unable to physically interact. In agreement with this notion, we have not yet been able to detect direct or indirect interactions between these proteins in DNA-binding studies.

Another unique feature of RuvA*_Mge_* is the mode in which this protein forms tripartite complexes with HJ resolvase RecU*_Mge_* and HJs. This is the first report to demonstrate an interaction between a member of the RecU protein family and a RuvA protein. RuvA*_Mge_* and RecU*_Mge_* were found to associate readily and stably on HJ substrates at a broad protein concentration range. In contrast, tripartite complexes of RuvA*_Eco_*, RuvC*_Eco_* and HJs were only observed at relatively low concentrations of RuvA*_Eco_*, because the latter protein has a higher affinity than RuvC*_Eco_* for HJ DNA [32]. At relatively high RuvA*_Eco_* concentrations, the HJ DNA will be saturated with protein, such that two RuvA*_Eco_* tetramers are bound to opposite faces of the junction. Thus, the binding of RuvC*_Eco_* to the junction is excluded [32], [33], [41]. At low RuvA*_Eco_* concentrations, however, the main protein-HJ complex that is formed is complex I, which consists of a single tetramer of RuvA_Eco_ bound to a single face of the junction. This structure may allow the binding of a RuvC*_Eco_* dimer to the other face of the DNA substrate, thereby generating a tripartite RuvAC*_Eco_*-HJ complex [32]. In analogy with this model, a tetramer of RuvA*_Mge_* bound to one side of a HJ may permit the binding of (multimers of) RecU*_Mge_* at the opposite side of the junction. Because RuvA*_Mge_* is unable to form stable octameric-HJ complexes, as discussed above, the tetrameric RuvA*_Mge_*-HJ complex may always be accessible, at one face of the junction, for binding by RecU*_Mge_*. This may explain why RecU*_Mge_* and RuvA*_Mge_* do not compete with each other for binding to HJs, but rather interact readily by forming a stable tripartite complex. This interaction does, however, lead to inhibition of the HJ resolution activity of RecU*_Mge_*, a phenomenon that parallels the inhibition of RuvC*_Eco_*-catalyzed HJ resolution by RuvA*_Eco_* [32]. It remains to be determined whether the RecU*_Mge_*-RuvA*_Mge_*-HJ complexes are stabilized exclusively by protein-DNA interactions or also by RecU*_Mge_*-RuvA*_Mge_* interactions; experiments aimed at the detection of such protein-protein interactions have hitherto not produced conclusive results. In addition, it is clear that the physiological role will have to be established of the RecU*_Mge_*-RuvA*_Mge_* interaction and the RuvA*_Mge_*-mediated inhibition of the HJ resolution activity of RecU*_Mge_*. Nevertheless, it is likely that a functional coupling exists between these proteins and that the combined activities of a complex of RuvB*_Mge_* and RuvA*_Mge_* may be linked to the resolvase activity of RecU*_Mge_*. Such a situation could be similar to that in *E. coli*, in which the RuvAB DNA branch migration complex is coupled to the RuvC resolvase in a RuvABC*_Eco_* resolvasome complex. In this regard, it is also interesting to note that a close association between RecU*_Mge_* and RuvA*_Mge_* (plus RuvB*_Mge_*) is also reflected in the genome of *M. genitalium*, in which the ORF encoding RecU*_Mge_* (MG352) is localized in the vicinity of the ORFs encoding RuvA*_Mge_* (MG358) and RuvB*_Mge_* (MG359).

Another issue that remains to be addressed is the nature of the four different RecU*_Mge_*-HJ complexes that were formed at relatively high concentrations of RecU*_Mge_*. In previous studies on this protein, only a single RecU*_Mge_*-HJ complex was observed due to the use of different DNA binding conditions [19], [20]. It was shown by protein crystallography and structure determination that the RecU homologues from *Bacillus subtilis* [42] and *Bacillus stearothermophilus* [43] exist as dimers. Based on this information, we speculate that the four RecU*_Mge_*-HJ complexes that were observed in this study consist of HJs bound by dimers, tetramers, hexamers and octamers, respectively, of RecU*_Mge_*. How the larger multimers would be accommodated on a single HJ, and how these would also leave room for binding of a RuvA*_Mge_* tetramer, which was observed for each of the four RecU*_Mge_*-HJ complexes, are challenging questions. The formation of large assemblies of proteins bound to a junction, however, is not unprecedented, as RuvA*_Eco_* mutant RuvA3m was reported to generate HJ-protein complexes consisting of six protein tetramers [35].

In conclusion, the studies of the RuvA, RuvB and RecU homologues from mycoplasmas have revealed that these proteins each have distinctive properties as opposed to their counterparts from other bacterial classes. It is possible that these unique features have emerged as a consequence of the evolutionary reduction that the genomes of the mycoplasmas are believed to have undergone. Specifically, the loss of a significant portion of an ancestral set of DNA recombination and repair enzymes may have required an accompanying modification of the function of the RuvA, RuvB and RecU proteins in order to preserve certain functionalities of the recombination and repair system. Nevertheless, the complete set of functions of this system in mycoplasmas is yet to be determined. In this regard, it is particularly interesting to learn how DNA recombination processes are achieved in *M. pneumoniae* in the absence of a functional RecU resolvase [19]. Although HJ resolvase activities may be exerted by other proteins, such proteins have not yet been identified in *M. pneumoniae*. Moreover, the lack of a functional RecU was proposed as a possible cause of the relatively low frequency of homologous DNA recombination events in *M. pneumoniae* [19]. Also, the HJ resolvase deficiency of *M. pneumoniae* may be associated with the difference between *M. pneumoniae* and *M. genitalium* in the specific mechanism by which homologous DNA recombination events occur in these species. In *M. genitalium*, the repeated DNA elements appear to recombine predominantly in a reciprocal fashion [7], [8], [12], whereas in *M. pneumoniae* such elements seem to recombine via a gene conversion-like mechanism, in which donor sequences are copied to the acceptor site and the original acceptor sequence is lost [9], [10], [11], [13], [14]. To address these and other issues related to the mechanism of homologous recombination in *M. pneumoniae* and *M. genitalium*, it is crucial that the entire set of putative DNA recombination and repair enzymes of these species be delineated. This will therefore be the goal of future studies.

## Materials and Methods

### Cloning of the *M. pneumoniae* MPN535 gene and *M. genitalium* MG358 gene

Bacterial DNA was purified from cultures of *M. pneumoniae* strain M129 (ATCC® no. 29342^TM^) and *M. genitalium* strain G37 (ATCC® no. 33530^TM^), as described previously [16], [44]. The MPN535 ORF of *M. pneumoniae* strain M129, which encodes a RuvA homologue, was amplified by PCR. The PCR reaction was performed using the following primers: RuvAmpn_fw (5′-GGTCGT*CATATG*ATTGCTTCAATTTTTGGAA-3′, which overlaps with the translation initiation codon [underlined] of MPN535) and primer RuvAmpn_rev (5′- GCAGCC*GGATCC* TTAGGCGGTTTTATTTGTAAC-3′, which overlaps with the antisense sequence of the translation termination codon [underlined] of the gene). The resulting 0.6-kilobase pairs (kb) PCR fragment was digested with *Nde*I and *Bam*HI (the recognition sites for these enzymes are indicated in italics in the sequences of primers RuvAmpn_fw and RuvAmpn_rev, respectively), and cloned into *Nde*I- and *Bam*HI-digested *E. coli* protein expression vectors, i.e. pET-11c and pET-16b (Novagen), generating plasmids pET-11c-RuvA*_Mpn_* and pET-16b-RuvA*_Mpn_*, respectively. Plasmid pET-11c-RuvA*_Mpn_* was used for expression of native RuvA*_Mpn_*, while plasmid pET-16b-RuvA*_Mpn_* was employed for expression of RuvA*_Mpn_* as an N-terminally poly histidine (H_10_)-tagged protein in *E. coli*.

Before cloning of the MG358 ORF of *M. genitalium* into *E. coli* protein expression vectors, a TGA codon within the ORF (encoding the Trp residue at position 27 of RuvA*_Mge_*) was changed into a TGG codon using a PCR-based mutagenesis procedure [19]. Following mutagenesis, MG358 was amplified by PCR using the primers RuvAmg_pETfw (5′-CGTCA*CATATG*ATTACATCTATCTTTGG -3′, which includes an *Nde*I restriction site [in italics] and the translation initiation codon of MG358 [underlined]) and RuvAmg_pETrv 5′-CGTCA*GGATCC*GGTATTAGGCGGTTTTATTTG-3′, which includes a *Bam*HI site [in italics] and the antisense sequence of the translation termination codon [underlined] of the gene). The 0.6-kb PCR product was digested with *Nde*I and *Bam*HI, and ligated into *Nde*I- and *Bam*HI-digested vectors pET-11c and pET-16b, resulting in plasmids pET-11c-RuvA*_Mge_* and pET-16b-RuvA*_Mge_*, respectively. These plasmids were used for expression of native and H_10_-tagged RuvA*_Mge_*, respectively, in *E. coli*. The integrity of all DNA constructs used in this study was checked by dideoxy sequencing, as described before [15].

### Protein expression and purification

The various pET-11c- and pET-16b-derived vectors were introduced into *E. coli* BL21(DE3) and the resulting strains were grown overnight at 37°C in LB medium containing 100 μg/ml ampicillin. The cultures were diluted 1∶100 in 300 ml LB medium with ampicillin and grown at 37°C to an optical density at 600 nm of 0.6. Protein expression was then induced by the addition of isopropyl-β-D-thiogalactopyranoside (IPTG) to a final concentration of 0.5 mM. After incubation for 2 hr at 30°C, the bacteria were harvested by centrifugation and stored at −20°C.

The H_10_-tagged RuvA*_Mpn_* and RuvA*_Mge_* proteins were both purified using the following protocol. Bacterial pellets were resuspended in 10 ml of buffer A (20 mM Tris-HCl pH 8.0, 1 M NaCl) containing 0.5 mg/ml of lysozyme. The suspension was sonicated on ice and clarified by centrifugation for 20 min at 12,000× g (at 4°C). To the supernatant, imidazole was added to a final concentration of 5 mM. Then, the supernatant was loaded onto a column containing 1 ml of Ni^2+^-nitroloacetic acid (Ni-NTA)-agarose (Qiagen), which was equilibrated previously in buffer A containing 5 mM imidazole. The column was washed with 5 ml of buffer A plus 5 mM imidazole and with 5 ml of buffer A plus 20 mM imidazole. The specifically bound proteins were eluted from the column with 8 ml of buffer A containing 250 mM imidazole. Fractions of 0.5 ml were collected, analyzed by SDS-polyacrylamide gel electrophoresis (SDS-PAGE), pooled, and dialyzed against a solution of 20 mM Tris-HCl (pH 7.4), 0.2 M NaCl, 0.1 mM EDTA, 1 mM DTT and 50% glycerol (buffer B). Aliquots of purified protein, which had an estimated homogeneity of >95%, were stored at −20°C.

The native RuvA proteins were purified by solubilization of the bacterial pellets in a buffer containing 20 mM Tris-HCl pH 7.5, 1 mM EDTA, 1 mM DTT and 0.5 mg/ml of lysozyme. After sonication and centrifugation (using similar procedures as described above), the RuvA proteins were precipitated with ammonium sulphate and resuspended in 20 mM Tris-HCl pH 7.4, 0.1 M NaCl, 0.1 mM EDTA, 1 mM DTT. The proteins were then subjected to affinity chromatography using Heparin Sepharose 6 Fast Flow (GE Healthcare). Proteins were eluted from the column material with a linear gradient from 0 M to 1 M NaCl in 20 mM Tris-HCl pH 7.4, 0.1 mM EDTA and 1 mM DTT. The RuvA-containing fractions were pooled, dialyzed against buffer B, and stored at −20°C.

The purifications of RecU*_Mge_*, RuvB_FH_ and RuvB*_Mge_* have been described before [18], [19], [20].

### SDS-PAGE

Proteins were separated by SDS-PAGE, as described by Laemmli [45]. Gels were stained with Coomassie brilliant blue (CBB), destained in 40% methanol/10% acetic acid, and recorded using a GelDoc XR system (Bio-Rad). Digital images were processed using Quantity One® 1-D Analysis Software (Bio-Rad).

### DNA substrates

The small DNA substrates that were used in the DNA binding experiments consisted of synthetic oligonucleotide substrates that were 5′ 6-FAM-labelled on a single strand. Holliday junction (HJ) substrate HJ 1.1, single-stranded oligonucleotide HJ11 and double-stranded substrate HJ11/HJ11rv have been described by Sluijter et al. [19]. Substrate HJ 1.1 is composed of the following four oligonucleotides: HJ11 (5′-GCGACGTGATCACCAGATGATTGCTAG-GCATGCTTTCCGCAAGAGAAGC-3′), HJ12 (5′-GGCTTCTCTTGCGGAAAGCATGCCTA-GCAATCCTGTCAGCTGCATGGAAC-3′), HJ13 (5′-GGTTCCATGCAGCTGACAGGATT-GCTAGGCTCAAGGCGAACTGCTAACGG-3′) and HJ14 (5′-ACCGTTAGCAGTTCG-CCTTGAGCCTAGCAATCATCTGGTGATCACGTCGC-3′). The sequence of oligonucleotide HJ11rv is 5′-GGCTTCTCTTGCGGAAAGCATGCCTAGCAATCATCTGGTGATCACGTC-GC-3′. The DNA helicase substrates (Fig. 6A) have been described in detail by Estevão and coworkers [18].

### DNA-binding assays

Binding of the RuvA proteins to various DNA substrates was carried out in 10-µl volumes and included 20 mM Tris-HCl pH 7.5, 1 mM DTT, 1 mM EDTA, 12.3 nM oligonucleotide substrate and various concentrations of RuvA proteins. After incubation on ice for 10 min, 1 μl was added of a solution containing 40% glycerol and 0.25% bromophenol blue. Then, the reaction mixtures were electrophoresed through 8% polyacrylamide gels in 0.5× TBE buffer (45 mM Tris, 45 mM boric acid, 1 mM EDTA). Following electrophoresis, the polyacrylamide gels were analyzed by fluorometry, using a Typhoon Trio™ 9200 Variable Mode Imager (GE Healthcare) in combination with the Typhoon Scanner Control v4.0 software (Amersham Bioscience). Images were processed using Quantity One® 1-D Analysis Software.

### Holliday junction (HJ) resolution assays

HJ resolution assays were carried out as described by Sluijter et al. [19]. Reactions were analyzed by electrophoresis through 12% polyacrylamide/1× TBE mini-gels. The relative RecU*_Mge_* (resolution) activity (Fig. 5C) was expressed as percentage of the protein's activity in the absence of RuvA*_Mge_*.

### DNA helicase and ATPase assays

DNA helicase assays were performed similarly as described before [18]. After deproteinization, the reactions mixtures were analyzed by electrophoresis through 12% polyacrylamide/1× TBE mini-gel and fluorometry. The ATPase activities of RuvB_FH_ and RuvB*_Mge_* were determined by using a β-nicotinamide adenine dinucleotide reduced form (NADH)-coupled assay on a VersaMax Tunable Microplate Reader (Molecular Devices) [15], [46].

## Supporting Information

Figure S1 **RuvA** ***_Mge_*** **is a tetramer in solution.** (A) Gel filtration analysis of RuvA*_Mge_*. Gel filtration chromatography was performed in a similar fashion as described previously [16], using a Sephadex G-150 column (length, 1.0 m; inner diameter, 1.0 cm). The column was run at 4 ml/h in 50 mM Tris-HCl (pH 7.5)/ 135 mM NaCl, and calibrated with blue dextran (2,000 kDa), bovine serum albumin (BSA, 66.4 kDa), ovalbumin (42.9 kDa), and cytochrome C (12.3 kDa). Fractions of 1.0 ml were collected and monitored by measuring the optical density at 280 nm (OD280, Y-axis at the left-hand side of the graph). The fractions eluted from a subsequent run, containing 15 µg of RuvA*_Mge_*, were precipitated with trichloroacetic acid, and separated on 12% SDS-PAGE gels. Gels were silver-stained and recorded using the GelDoc XR system. RuvA*_Mge_* was quantified by densitometry using Quantity One® 1-D Analysis Software (Bio-Rad). The relative concentration of RuvA*_Mge_* (Y-axis on the right-hand side, in arbitrary units) is shown for column fractions 23 to 39. In all other fractions, RuvA*_Mge_* was not detected. (B) Calibration curve obtained from the gel filtration experiment shown in (A). The molecular weight of protein size standards (♦) is plotted against the elution volume (V_e_) divided by the void volume (V_0_) of the column (V_e_/V_0_). V_0_ was determined with blue dextran. The V_e_/V_0_ of RuvA*_Mge_* is marked on the calibration curve (×).
(TIF)
