# Supplementary figures and images for: The RuvA Homologues from Mycoplasma genitalium and Mycoplasma pneumoniae Exhibit Unique Functional Characteristics
Source: PLoS One. 2012 May 30;7(5):e38301. doi: 10.1371/journal.pone.0038301 (PMC3364216; doi:10.1371/journal.pone.0038301)

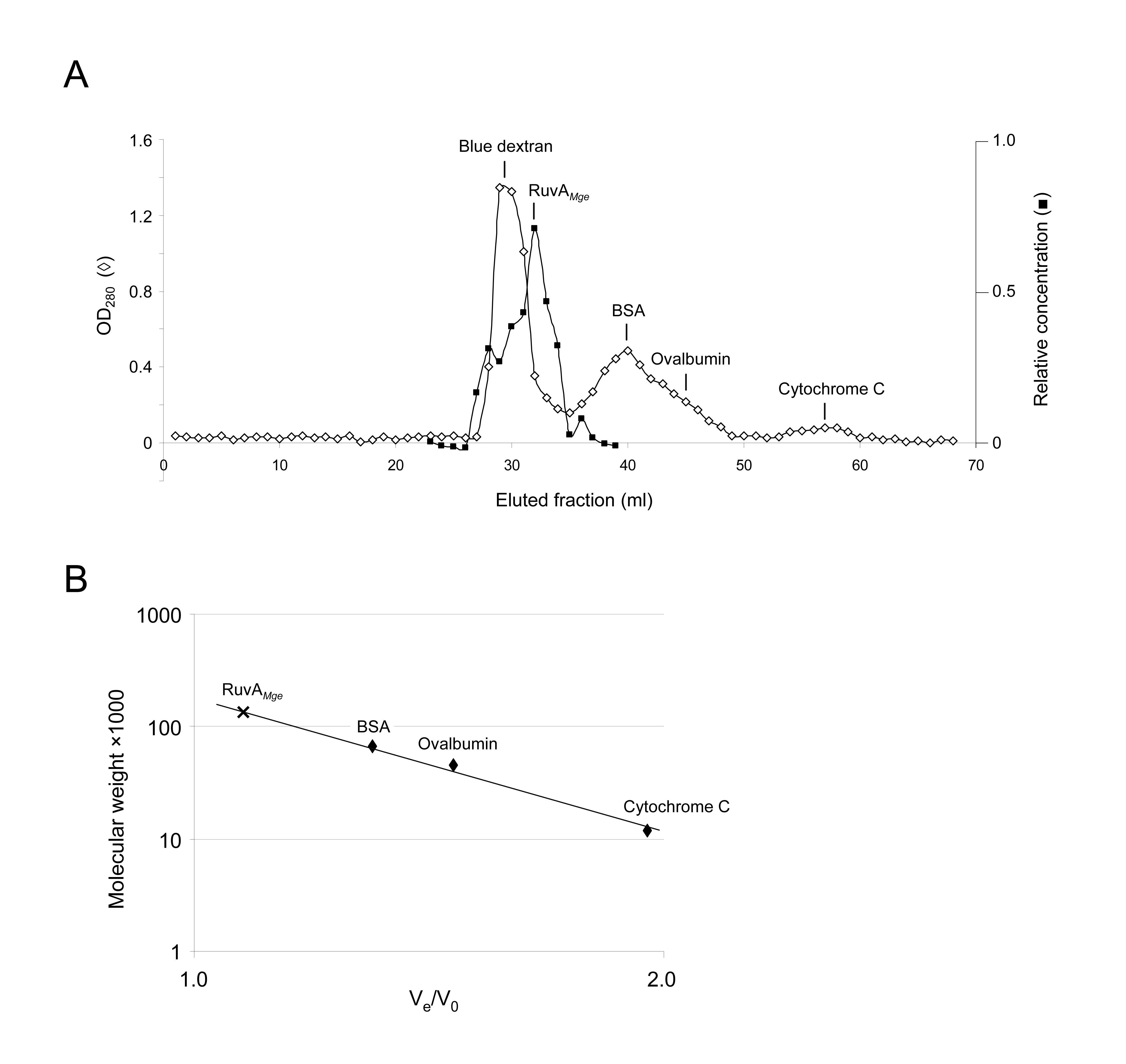

Supplement: Figure S1 — RuvA Mge is a tetramer in solution. (A) Gel filtration analysis of RuvAMge. Gel filtration chromatography was performed in a similar fashion as described previously [16], using a Sephadex G-150 column (length, 1.0 m; inner diameter, 1.0 cm). The column was run at 4 ml/h in 50 mM Tris-HCl (pH 7.5)/ 135 mM NaCl, and calibrated with blue dextran (2,000 kDa), bovine serum albumin (BSA, 66.4 kDa), ovalbumin (42.9 kDa), and cytochrome C (12.3 kDa). Fractions of 1.0 ml were collected and monitored by measuring the optical density at 280 nm (OD280, Y-axis at the left-hand side of the graph). The fractions eluted from a subsequent run, containing 15 µg of RuvAMge, were precipitated with trichloroacetic acid, and separated on 12% SDS-PAGE gels. Gels were silver-stained and recorded using the GelDoc XR system. RuvAMge was quantified by densitometry using Quantity One® 1-D Analysis Software (Bio-Rad). The relative concentration of RuvAMge (Y-axis on the right-hand side, in arbitrary units) is shown for column fractions 23 to 39. In all other fractions, RuvAMge was not detected. (B) Calibration curve obtained from the gel filtration experiment shown in (A). The molecular weight of protein size standards (♦) is plotted against the elution volume (Ve) divided by the void volume (V0) of the column (Ve/V0). V0 was determined with blue dextran. The Ve/V0 of RuvAMge is marked on the calibration curve (×). (TIF) [file pone.0038301.s001.tif]
